# Development and Validation of a Novel *Leishmania donovani* Screening Cascade for High-Throughput Screening Using a Novel Axenic Assay with High Predictivity of Leishmanicidal Intracellular Activity

**DOI:** 10.1371/journal.pntd.0004094

**Published:** 2015-09-25

**Authors:** Andrea Nühs, Manu De Rycker, Sujatha Manthri, Eamon Comer, Christina A. Scherer, Stuart L. Schreiber, Jean-Robert Ioset, David W. Gray

**Affiliations:** 1 Drug Discovery Unit, Division of Biological Chemistry and Drug Discovery, University of Dundee, Dundee, United Kingdom; 2 Broad Institute, Cambridge, Massachusetts, United States of America; 3 Drugs for Neglected Diseases initiative, Geneva, Switzerland; Pasteur Institute, FRANCE

## Abstract

Visceral leishmaniasis is an important parasitic disease of the developing world with a limited arsenal of drugs available for treatment. The existing drugs have significant deficiencies so there is an urgent need for new and improved drugs. In the human host, *Leishmania* are obligate intracellular parasites which poses particular challenges in terms of drug discovery. To achieve sufficient throughput and robustness, free-living parasites are often used in primary screening assays as a surrogate for the more complex intracellular assays. We and others have found that such axenic assays have a high false positive rate relative to the intracellular assays, and that this limits their usefulness as a primary platform for screening of large compound collections. While many different reasons could lie behind the poor translation from axenic parasite to intracellular parasite, we show here that a key factor is the identification of growth slowing and cytostatic compounds by axenic assays in addition to the more desirable cytocidal compounds. We present a screening cascade based on a novel cytocidal-only axenic amastigote assay, developed by increasing starting density of cells and lowering the limit of detection, and show that it has a much improved translation to the intracellular assay. We propose that this assay is an improved primary platform in a new *Leishmania* screening cascade designed for the screening of large compound collections. This cascade was employed to screen a diversity-oriented-synthesis library, and yielded two novel antileishmanial chemotypes. The approach we have taken may have broad relevance to anti-infective and anti-parasitic drug discovery.

## Author Summary

New drugs for visceral leishmaniasis, are urgently required as existing drugs have serious shortcomings including toxicity and drug resistance. This disease is caused by parasites from the *Leishmania* family which live inside human cells. Screening large collections of chemicals (>100,000) to identify compounds that kill parasites has been used to identify new start points for drug discovery. It is complex and expensive to look at such numbers using intracellular parasites. To circumvent this, many groups screen using parasites adapted to grow outside human cells (axenic forms). However, the established protocols identify growth slowing compounds as well as compounds that kill parasites. Cytocidal compounds are better start points for drug discovery. Here we present a screening cascade based on a modified axenic *Leishmania* assay adapted to identify compounds that kill parasites. We show that these compounds have a higher probability of being active against intracellular parasites. This new screening cascade was used to screen a compound collection and led to the identification of two new chemical series with antileishmanial activity. Their activity was confirmed against intracellular parasites. They are potential candidates for further drug development. The approach we have taken may have broad relevance to anti-infective and anti-parasitic drug discovery.

## Introduction

The protozoan parasites of the genus *Leishmania* are the causative agents of leishmaniasis, a group of diseases that is prevalent in 98 countries and 3 territories with approximately 1.3 million new cases occurring annually. There are estimated to be 20,000 to 40,000 deaths per year [1]. Leishmaniasis occurs in three main forms. Visceral leishmaniasis is the most severe form, where the parasites migrate to the internal organs, particularly the spleen and liver, resulting in death if untreated [2]. Cutaneous and mucocutaneous leishmaniasis are dermal infections which carry lower mortality but are highly disfiguring. The associated social stigmatization can have significant negative effects on psychological well-being [3, 4].

Currently, treatment of visceral leishmaniasis is limited to a few drugs used either in monotherapy or in combination. For reasons still to be understood, some of the treatments show a lack of clinical efficacy in certain geographic regions, notably in East Africa. In India and Nepal the emergence of resistance to antimonial therapy also limits the treatment options. Additionally, the occurrence of moderate to severe adverse effects, the existence of contraindications such as pregnancy in the case of miltefosine, the relatively high cost of the treatments as well as the logistical complexity related to the storage and use of drugs in the endemic regions further restrict the use of amphotericin B, miltefosine, antimonials and paromomycin. There is therefore an urgent need for new and better drugs to address treatment needs [5–7].

High-throughput screening of diverse compound sets in phenotypic assays has proved an effective way of discovering new start points for drug discovery [8, 9]. To facilitate screening of large compound collections against *Leishmania*, axenic amastigotes have been used as a surrogate for the disease-causing intracellular form [10–12]. Axenic amastigotes are thought to be a more relevant model of the human lifecycle stages of leishmania infection in comparison to the promastigote [13–17]. Several assays using axenic stages of *Leishmania* have been developed to the scale and robustness appropriate for library screening. However, we and others have questioned the relevance of these assays due to the poor translation of many axenic hit molecules into the physiologically more relevant but more complex intracellular *Leishmania* assays [10, 18–22].

The poor confirmation rate of promastigote and axenic amastigote active compounds in intracellular assays could result from the many differences between the assays, including the localisation of intracellular parasites in the more difficult to access parasitophorous vacuole, differences in pH and composition of the growth media, stage-specific differences such as alternate energy pathway usage [16, 23, 24], etc. Insights derived from our work with *Trypanosoma brucei* [25] point to another potential factor: many published axenic assays are likely to report not only cytocidal compounds but also growth slowing and cytostatic compounds. The latter two types of compounds are unlikely to show activity in intracellular assays which, due to the slow replication rate of the intracellular parasites and sensitivity of high content readers, tend to only report cytocidal molecules.

In this paper we report the development and validation of a novel axenic amastigote *Leishmania* assay that reports only cytocidal molecules. This new assay was validated using a diverse compound library previously screened in our historic axenic format and in the intracellular assay [18]. We also used the new axenic assay as a primary screening platform to screen a diversity-oriented synthesis library [26]. We identified two new chemical series with antileishmanial activity and show that the new axenic assay has a significantly improved translation to the intracellular assay. We propose that the novel axenic assay provides *Leishmania* drug discovery efforts with an improved high-throughput platform for the screening of large compound libraries as it does not suffer from the high false positive rates seen in other axenic assays and provides both higher throughput and better robustness than intracellular assays.

## Methods

### Parasite strain and maintenance

*Leishmania donovani* BOB cells (LdBOB) used in this study are a cloned line from strain MHOM/SD/62/1S-CL2 [13]. Cultures were maintained as described previously [18].

### Materials

White, clear-bottom assay plates (384-wells) were obtained from Greiner (historic axenic assay) and Corning (novel axenic assay). Echo plates were obtained from LabCyte.

### Chemicals

Amphotericin B, Dimethyl Sulfoxide (anhydrous, ≥ 99.9%, DMSO) and Resazurin were obtained from Sigma. The small diverse library used in this study contained 15,667 compounds, dissolved at 10 mM in DMSO and stored under low-oxygen and low-humidity conditions. The design of this diverse library is described elsewhere [27]. The Diversity-Orientated Synthesis (DOS) Informer Set (9,907 compounds) is a subset of the Broad Institute’s diversity-oriented synthesis library, which comprises approximately 100,000 structurally diverse small molecules that combine the complexity of natural products and the efficiency of high-throughput synthesis [26, 28, 29]. These small molecules have a higher ratio of sp^3^-hybridized atoms and stereocentres relative to compounds found in conventional screening collections [30]. The compounds are structurally diverse, from >30 different individual libraries and >250 unique scaffolds. Moreover, where possible, all stereoisomers have been individually synthesized, providing rich stereo-structure-activity relationship (SSAR) data directly from primary screens and facilitating rapid prioritization and optimization of hit compounds.

### Compound handling

Compounds were dispensed into 384-well assay plates by acoustic dispensing (LabCyte ECHO). For potency determinations, ten-point one in three dilution curves were generated, with a top concentration of 50μM. Potencies are reported as pEC_50_ (-LOG(EC_50_[M])).

### Data analysis

All data was processed using IDBS ActivityBase. Raw data was converted into percent inhibition through linear regression by setting the high inhibition control as 100% and the no inhibition control as 0%. For primary single concentration screening in the novel axenic assay we introduced a static control as 0% inhibition (signal at time of assay start), which is explained in more detail in the results section. Potency plates were normalised to DMSO control (0% effect) and 2μM amphotericin B control (100% effect). Quality control criteria for passing plates were as follows: robust z’ ≥ 0.5, signal to background > 3,% coefficient of variation for 0% inhibition controls < 15. The formula used to calculate robust z’ is 1-((3 x (1.4826 x MAD [0% inhibition controls] + 1.4826 x MAD [100% inhibition controls]))/(Median [0% inhibition controls]—Median [100% inhibition controls]), with MAD the median absolute deviation. The formula used for signal to background is: Median [0% inhibition controls] / Median [100% inhibition controls]. Curve fitting was carried out using the following 4 parameter logistic equation: y = A + (B - A) / (1 + ((10^C^) / x)^D^), where A =% inhibition at bottom, B =% inhibition at top, C = 50% effect concentration (EC_50_), D = slope, *x* = inhibitor concentration and y =% inhibition. For compounds with low activity and poor definition of the curve top, B was fixed to 100. For the determination of the reference compound panel potency, all experiments were carried out with a minimum of three independent repeats.

### Limit of detection (LoD)

Serial dilutions of LdBOB cells with defined cell concentrations were made. Each of the resulting concentrations was dispensed into at least 24 wells of a 384-well plate (50 μl and 25 μl per well for determining the LoD of the historic and novel axenic assay respectively), the rest of the wells contained media only (blank). The read-out for the historic axenic assay was performed by adding resazurin at 0.05 mM final concentration followed by incubation for 4 h at 37°C and 5% CO_2_. Fluorescence intensity was then measured using a Perkin Elmer Victor 3 plate-reader (excitation 528 nm, emission 590 nm). The read-out for the novel axenic assay was carried out by adding BacTiter-Glo (Promega) (volume equal to culture media volume) to each well and the luminescence was immediately read in a Victor 3 plate-reader. A linear regression was fitted and the LoD was derived as the number of cells equal to the mean signal of the blank wells plus 3 times the standard deviation. The values were determined from 4 independent experiments LoDs are reported as LoD +/- Standard Deviation (StDev).

### High-throughput assays

#### Historic axenic assay

The assay conditions are described previously [18]. Briefly LdBOB amastigote-like cells were seeded at 250 cells per well in a 384-well plate containing the compounds. After a 68 h incubation at 37°C and 5% CO_2_, resazurin was added at 0.05 mM final concentration. Plates were incubated for a further 4 h and then fluorescence was measured (excitation 528 nm, emission 590 nm) with a plate reader.

#### Intramacrophage assay

The assay conditions are described previously [18]. Briefly PMA differentiated THP-1 cells were infected overnight with eGFP expressing LdBOB amastigote-like cells at a multiplicity of infection of 5. Next, any remaining free amastigote-like cells are removed and compounds are added. The microscopy-based read-out is done after a four day compound incubation.

#### HepG2 assay

The assay conditions are described previously [31]. Briefly HepG2 cells were incubated for 72 h with compounds, followed by a resazurin-based read-out (fluorescence, excitation 528 nm and emission 590 nm) with a plate reader.

#### Novel axenic assay

Test compounds were pre-dispensed into white 384 well plates (Corning). For library screens one compound per well was tested at the indicated final concentration. The following controls were included on the plates: maximum effect control: Amphotericin B (final concentration 2 μM), ATP contamination control: media (± DMSO), cell growth control: DMSO, zero percent effect control: cells at starting concentration. The zero percent effect control wells are left empty during the course of the assay. Media only was dispensed into the ATP-control wells followed by dispensing LdBOB axenic amastigote-like cells at 2 x 10^4^ cells / well to the rest of the wells by using a *Well*mate Microplate Dispenser. The assay volume is 25 μl / well. After 72 ± 3 h at 37°C and 5% CO_2_ LdBOB axenic amastigote-like cells are added to the zero percent effect control wells at 2 x 10^4^ cells / well followed by read-out with “BacTiter-Glo Microbial Cell Viability Assay” from Promega according to manufacturer’s instructions. Plates were then sealed with clear film and relative luminescence was detected using a plate reader (Victor 3) from Perkin Elmer or PHERAstar FS from BMG LABTECH, with 0.5 s detection time per well). Potency determinations were carried out as library screens with the exception of the following: ten-point curves with one in three dilutions were generated with a top concentration of 50 μM.

## Results

### Novel axenic assay development

**Fig 1:**
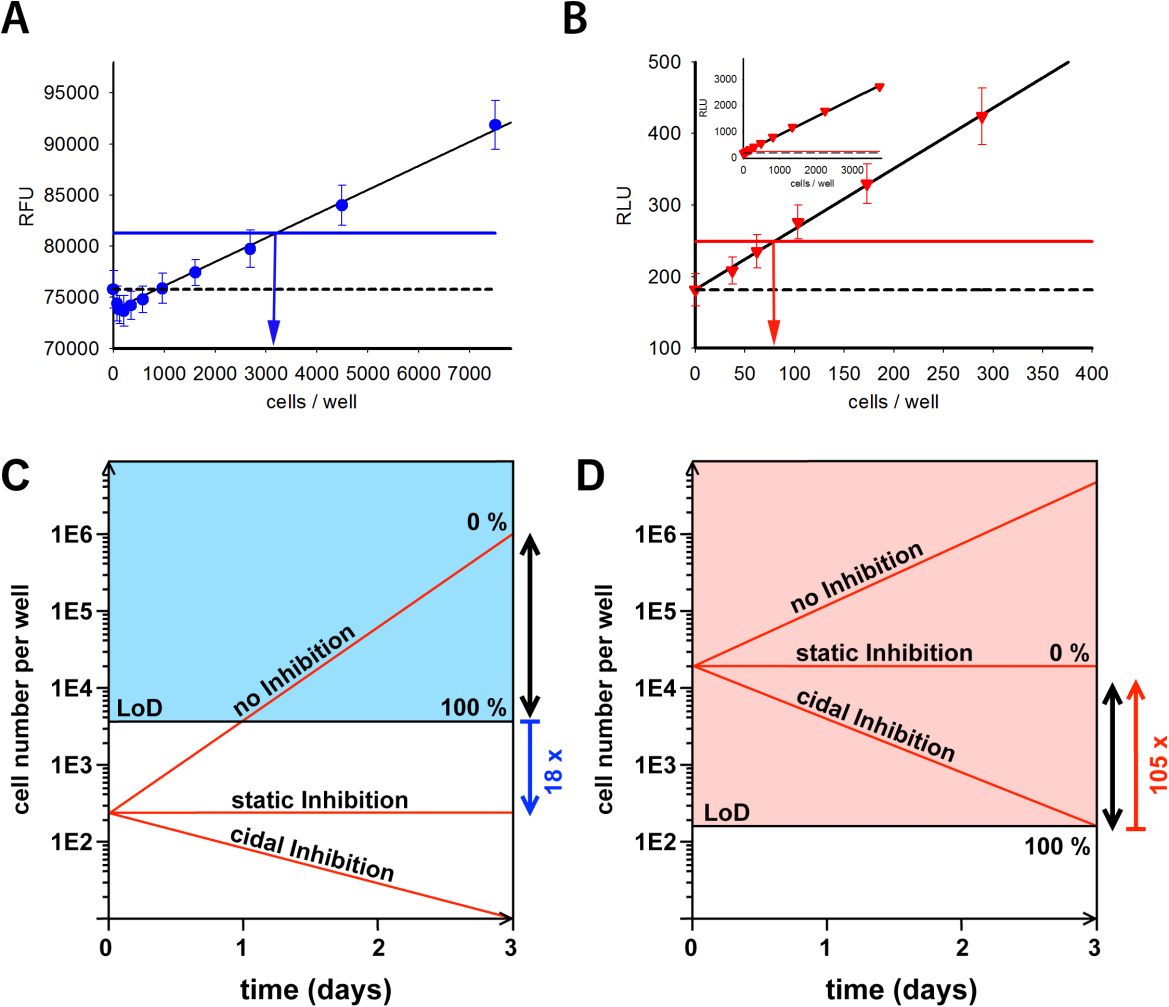
Limit-of-detection of historic and novel axenic assay and assay models. A: Detection limit of the historic axenic assay. Black line shows the linear regression (R^2^ = 0.983, p < 0.0001). Dashed line shows average value of blanks. Blue line shows detection limit (3x standard deviation above the blanks value) for this assay. Vertical blue arrow indicates the number of cells at the limit of detection in this single experiment. Data is from a representative experiment of 4 with a minimum of 24 technical replicates. B: Detection limit of the novel axenic assay. Black line shows the linear regression (R^2^ = 0.998, p < 0.0001). Dashed line shows average value of blanks. Red line shows detection limit for this assay (3x standard deviation above the blanks value). Red vertical arrow indicates the number of cells at the limit of detection in this single experiment. Data is from a representative experiment of 4 with a minimum of 24 technical replicates. Inset shows data from a similar range of cell densities as used in 1A. C and D: The coloured areas represent cell densities that can be detected with the respective assay formats (C: historic axenic assay, D: novel axenic assay). Red lines represent different cell-growth inhibition scenarios during the course of this assay: compounds that do not inhibit cell growth (marked “no Inhibition”), compounds that arrest cell growth, without killing cells (marked “static Inhibition”), and compounds that kill cells (marked with “cidal Inhibition”). The black double arrows represent the analysis window. Blue (historic assay) and red (novel assay) arrows show the fold difference between the starting density and detection limit.

The limit of detection (LoD) of the resazurin readout is 4,390 ± 1,980 cells per well; N = 4 (Fig 1A). The starting density (250 cells per well) of the historic axenic assay is therefore 18-fold below the limit of detection (modelled in Fig 1C). Consequently, compounds that either stop or inhibit growth below 4,390 cells within the 72 hour incubation period, but which do not kill the parasites, will look identical to truly cytocidal compounds.

The limit of detection of the BacTiter-Glo readout is substantially lower at 190 ± 110 cells per well; N = 4 (Fig 1B). While this is below the 250 cell starting density of the historic axenic assay, it does not give a sufficiently robust signal window to differentiate truly cytocidal compounds. To achieve this, we increased the starting cell density 80-fold to 20,000 cells/well.

We also changed the data analysis method by modifying the normalisation procedure so that 0% inhibition reflects no growth of the parasites. To obtain the no growth control measurement, 20,000 cells were seeded in plates (the starting assay density) immediately before addition of the BacTiter-Glo reagent. Amphothericin B, a known fast and cidal acting compound, was used to define the 100% effect. The technology switch from resazurin to BacTiter-Glo, cell number increase and change in normalisation combine to produce a robust screening protocol with a signal window of 105-fold of the LoD. This large window allows clear differentiation between cytocidal and growth slowing / static compounds (Fig 1D). A comparison of the main parameters that differ between both axenic assays is shown in Table 1.

**Table 1:** Comparison of the novel versus the historic axenic *Leishmania donovani* assay.

Parameter	Novel axenic	Historic axenic
Starting cell density	20,000 cells / well	250 cells / well
Read-out (Substrate)	luminescent (BacTiter-Glo)	fluorescent (Resazurin)
Limit of detection	190 (110) cells	4,390 (1,980) cells
0% Effect	starting density	endpoint density
100% Effect	2 μM Amphotericin B	media only

Numbers in brackets indicate the standard deviation.

### Assay performance

During assay validation of the single concentration screening format, 155 384-well plates were screened. The assay proved to be robust with an average robust Z-factor of 0.88 (± 0.04 StDev) and a Signal to Background ratio of 18.7 (± 4.6 StDev). Robustness was further confirmed by the absence of significant effects on assay performance when varying assay starting day, cell stock used or cell passage number used (Fig A-C in S1 Text). Two independent replicates of a set of 35 compounds in potency mode demonstrated very good reproducibility (R^2^ = 0.95, Fig D in S1 Text)). Amphotericin B was included as a control on each plate, and its potency was reproducible from run to run over an extended time period, further confirming the robustness of the assay (average pEC_50_ of 6.9 (± 0.1 StDev)). Overall the performance indicators support that the assay presented here is suitable for high-throughput screening.

### Comparison with historic axenic and intramacrophage assay

**Fig 2:**
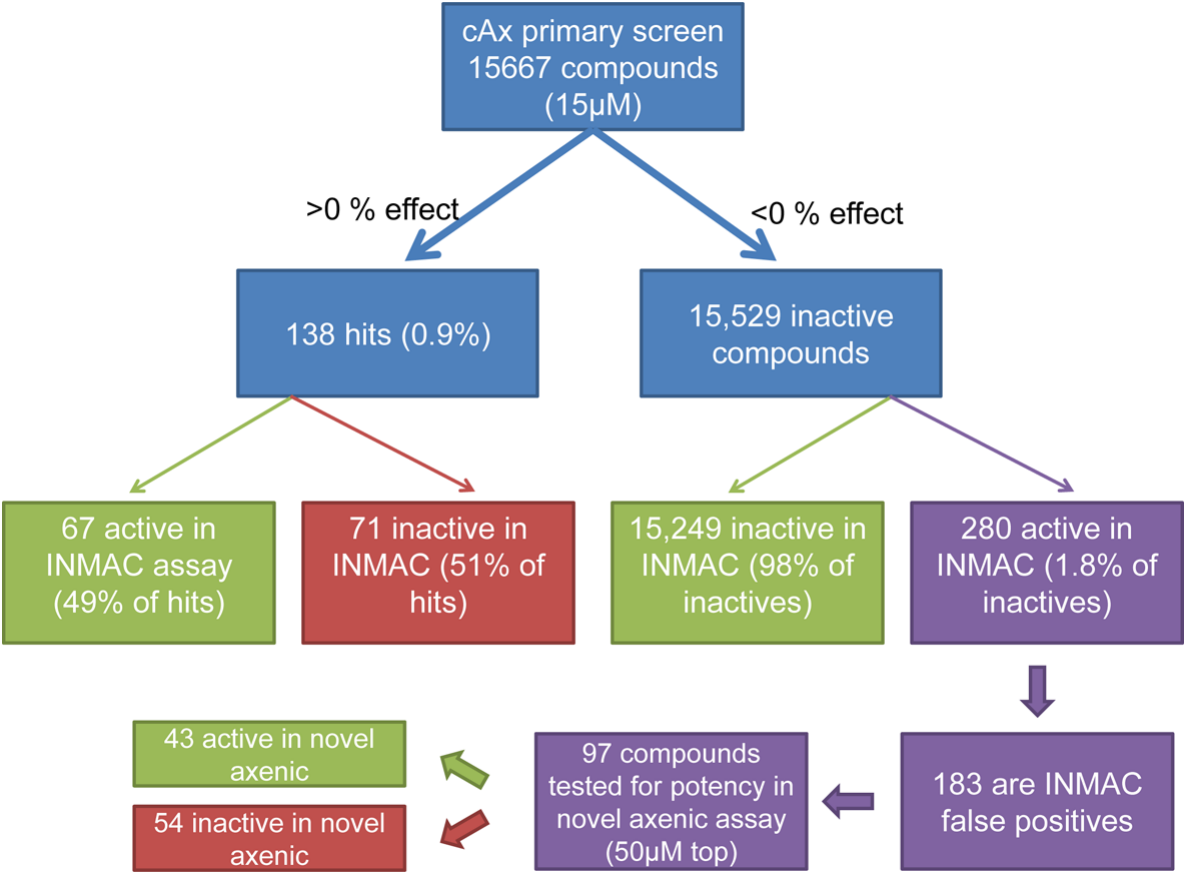
Library screen with novel axenic assay, comparison with intracellular results. 15,667 compounds were screened in the novel axenic assay at 15μM. Comparison with the intracellular assay shows that 67 of the hits are also active in the intracellular assay whereas 71 of the hits are not. 280 novel axenic assay inactive compounds were hits in the intracellular assay, 170 of these compounds showed toxicity in the intracellular assay (in single point mode, at 50μM, or less than 3-fold selectivity window in potency assay) and are therefore considered false positives in the intracellular assay. Potencies were determined for 110 non-toxic compounds in the novel axenic and intracellular assays. 13 compounds were inactive in the intracellular assay and are added to the false positive count (13 + 170 = 183). 97 compounds had confirmed activity in the intracellular assay; 42 of these compounds showed activity in the novel axenic assay, while 54 did not. (INMAC = intracellular assay). Hit criteria as described in Table A in S1 Text.

We previously screened a diverse compound set with around 16,000 compounds in the historic non-cidal axenic assay as well as in an intramacrophage assay [18]. The axenic assay identified 378 compounds that were active at 3μM (2.4% hit-rate), with only a relatively small proportion (83 compounds, 22% of the hits) also active in the intracellular assay (at 50μM). We reported that this high false-positive rate was the main impediment to using the axenic assay as a primary screening platform and proposed that a significant part of the high false-positive rate might be due to the identification of growth slowing and cytostatic compounds. To evaluate this, we re-screened this set of compounds in the novel axenic assay (at 15μM) and analysed the data using the intramacrophage assay as the gold standard (at 50μM) (Fig 2). The hit criteria and controls used for each assay are summarised in Table A in S1 Text. In contrast to the high hit-rate seen in the historic assay the novel assay only reported 138 hits (0.9% hit-rate), in spite of screening at a 3-fold higher concentration. Relative to the historic axenic assay, a much larger fraction of the novel axenic assay hits showed activity in the intracellular assay (49% of novel axenic hits are active in intracellular assay versus 22% for the historic assay). The analysis also showed that only 22% of the compounds active in the historic axenic assay were also active (i.e. cidal) in the novel assay, indicating that a large fraction of the compounds identified by the historic assay do not actually kill the parasites.

A significant number of compounds (280) showed activity in the intracellular assay at 50μM but were inactive in the novel axenic assay screen at 15μM. However, a large fraction of these compounds (170) owe their intracellular assay activity to toxicity against the THP-1 host cells. As such, they should be considered false positives in the intracellular assay rather than false negatives in the novel cidal axenic assay.

As a higher screening concentration was used in the intracellular assay we sought to determine whether this difference could account for the lack of axenic activity for the false negatives. To do so we determined the potency of 110 non-toxic, apparent false-negative compounds in the novel axenic and INMAC assays using the same top concentration (50 μM). A small number of compounds were inactive when tested in potency mode in the intracellular assay (13) which brings the tally for false positives to 183. Just under half of the compounds tested (43) now showed activity in the novel axenic assay. The remaining false-negative compounds had weak activity in the intracellular assay with 51 compounds having a pEC_50_ < 5 (EC_50_>10μM) and 3 compounds with a pEC_50_ between 5 and 5.4.

### Potency comparison of compounds in novel axenic and intramacrophage assay

**Fig 3:**
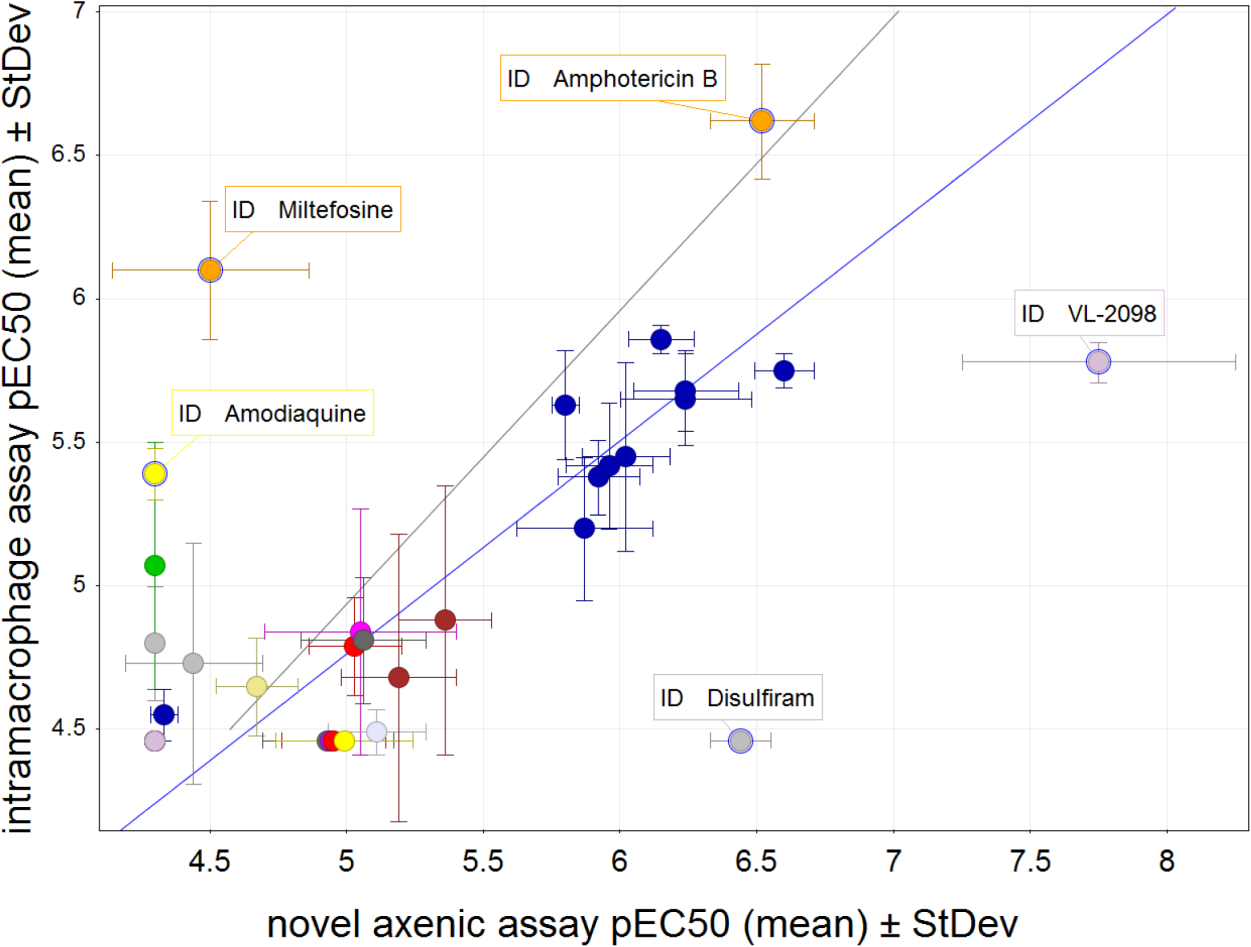
Potency comparison of a series of structurally distinct compounds between the novel axenic and intramacrophage assay. In this scatterplot the mean pEC_50_ values for control compounds representing a range of pharmacophores are compared between the novel and intramacrophage assay. Data set represents three or more replicates and the standard deviation is shown with error bars for each assay. Black line shows equipotency between both assays, blue line shows linear regression for compounds that are active in both assay (with outliers miltefosine and VL-2098 excluded, R^2^ = 0.81).

We determined the potency of a panel of 36 selected control compounds in the novel axenic and intramacrophage assays (Table B in S1 Text and Fig 3). This panel includes drugs used in the field for the treatment of visceral leishmaniasis (amphotericin B, miltefosine and paromomycin), representative compounds of chemical classes currently under preclinical or clinical development including nitroimidazoles and oxaboroles as well as a few earlier stage hits. The majority of these compound series show activity in animal models. In total, 21 compounds showed activity in both the novel axenic assay and the intracellular assay, and 7 compounds were inactive in both assays at the concentrations tested. With the exception of two compounds (miltefosine and VL-2098), the compounds that were active in both assays exhibited a good correlation between the two *Leishmania* assays (R^2^ = 0.81). Of the 8 compounds that were only active in one of the two assays most (5) were weakly active (pEC_50_<5). There were examples of compounds showing higher potency in the cidal axenic assay (disulfiram and VL-2098) than the intracellular assay and vice versa (miltefosine and amodiaquine). However, all these compounds would have been defined as active using the cidal axenic assay i.e. they would not have been missed.

### Screening cascade

**Fig 4:**
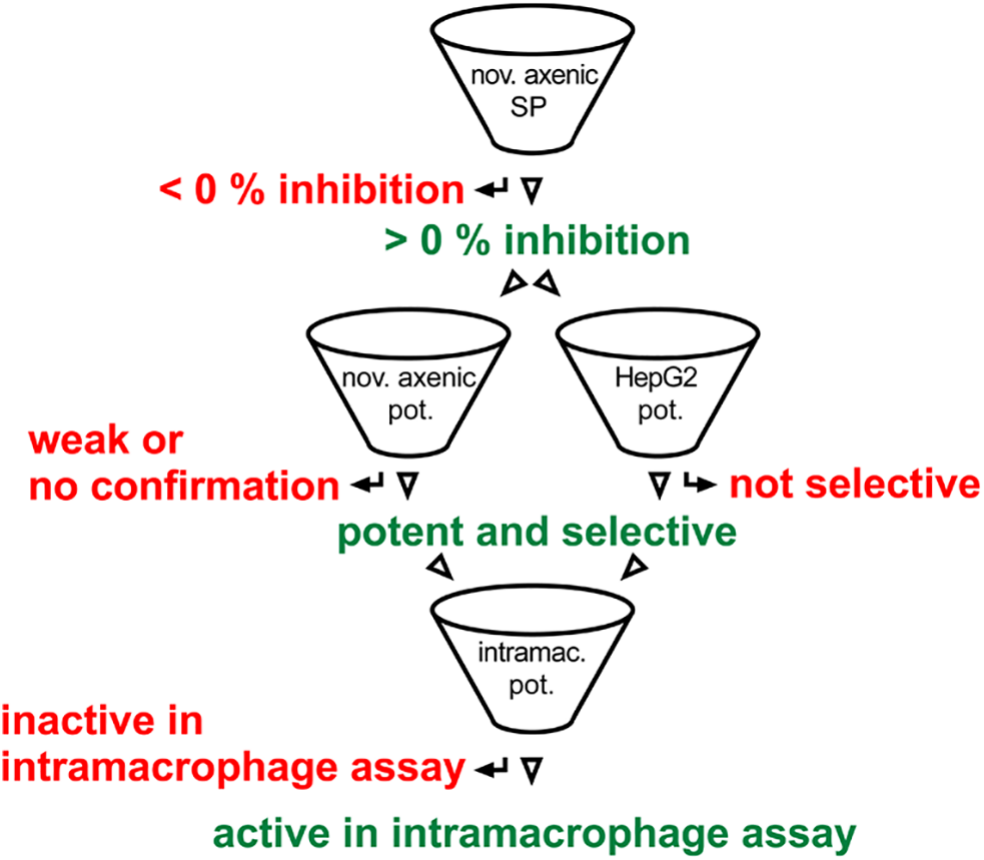
Screening cascade. Novel axenic assay is primary, single concentration (SP) entry to the cascade, followed by confirmation, assessment of potency and selectivity using the novel axenic assay and a mammalian counterscreen assay (HepG2) in potency mode (pot.). Potent and selective hits are then profiled in the intramacrophage assay.

Our results show that the novel axenic assay is more suitable for high-throughput screening than both our historic axenic assay and the intracellular assay. Relative to the historic axenic assay it detects many less false positives, and relative to the intracellular assay it provides much higher throughput and robustness (summarised in Table 2). We therefore propose a new *Leishmania* hit-discovery screening cascade that uses the novel axenic assay as primary single point assay (Fig 4). In a semi-manual format, a single scientist can run 60 plates per week giving a capacity to screen 20,000 test compounds weekly. The throughput can be scaled further using additional automation or scientists. Hits from the primary screen may be generically toxic and should be followed up with potency determinations in both the novel axenic assay and a mammalian counterscreen (we use HepG2 cells). Compounds with suitable activity and a selectivity window can then be taken forward to the intracellular assay for further confirmation of activity.

**Table 2:** Suitability of the *Leishmania donovani* assays for diversity screening.

	Historic axenic	Novel axenic	Intramacrophage
Predictivity (relative to intramacrophage activity)	Poor	Improved	N/A
Throughput	> 60 plates / week	> 60 plates / week	~ 20 plates / week
Assay duration	3 days	3 days	9 days
Robustness	High	High	Medium
Equipment	standard screening equipment	standard screening equipment	Highly specialised equipment
Key issues	High false positive rate	Expensive read-out reagent	High threshold to detect hits (many developable series may be left unidentified). Labour intensive and lack of robustness.

### DOS library screen

**Fig 5:**
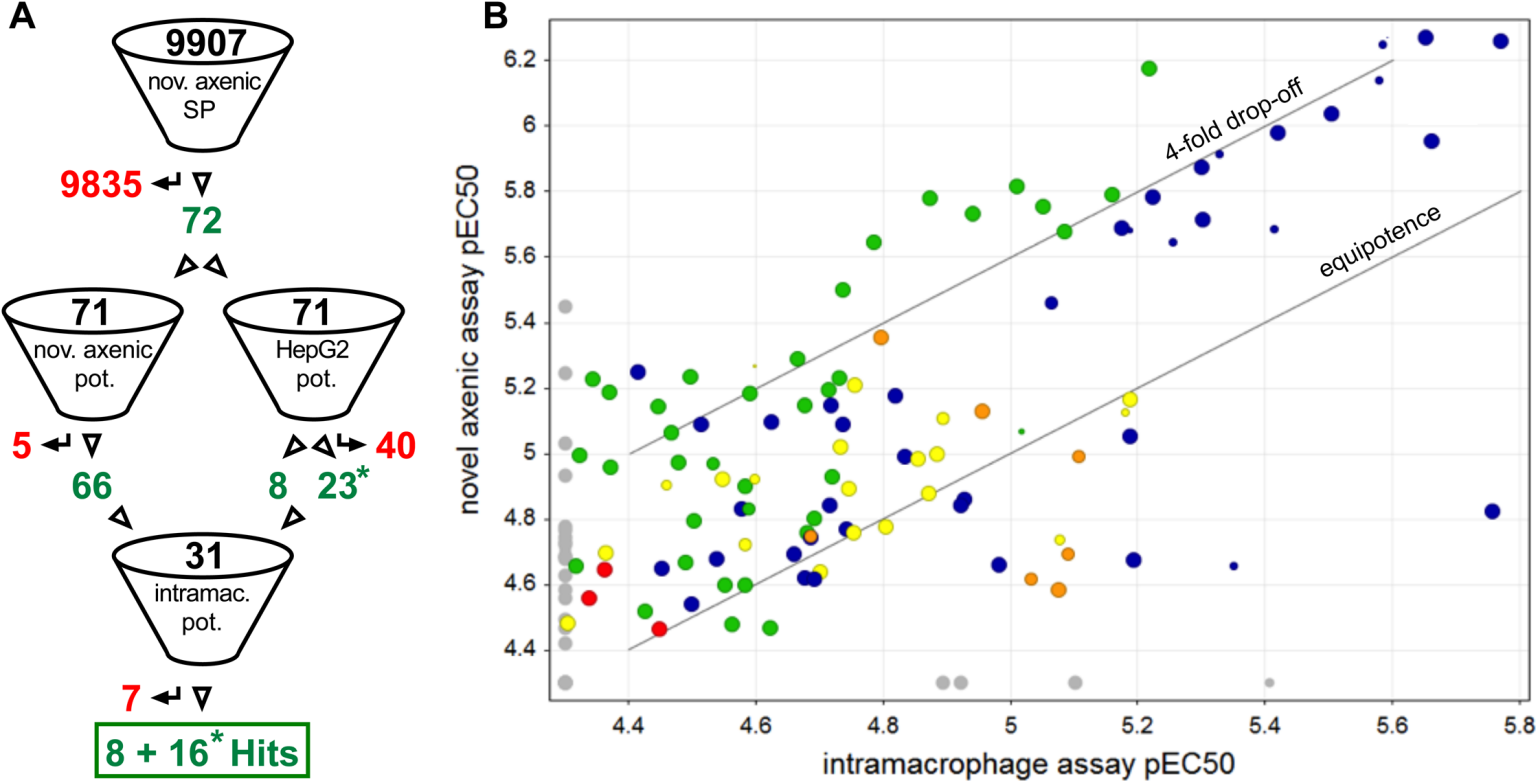
Screening cascade for DOS screen (A) and potency comparison of hits from the DOS library between the novel axenic and intramacrophage assay (B). A: Screening cascade. Numbers of compounds that passed and failed the hit selection criteria are shown respectively in green and red. Numbers marked with an asterisk indicate that the selectivity window is unknown and may be ≥ 10 (no effect seen in HepG2 assay, activity in the cidal axenic assay pEC50<5.3). The cascade starts with screening 9,907 compounds in the novel axenic assay at a single concentration (nov. axenic SP). Identified hits are processed in potency format in both the novel axenic assay (axenic pot., for activity confirmation) and HepG2 assay (HepG2 pot., toxicity information against the human cell line HepG2). Active compounds that provide a ≥ 10-fold toxicity window are processed in the intramacrophage potency assay (intramac. pot.) for hit confirmation, resulting in 24 hits. B: Comparison of novel axenic and intramacrophage mean pEC_50_ values for 141 compounds. The size of the markers is inversely related to the toxicity against the THP-1 cells (i.e. high toxicity–small symbol). Colour is by series (green: Ortho Azetidine Nitrile, blue: Azetidine Nitrile, orange: Povarov, red: SnAr 8-ortho, yellow: other). Data set represents three biological replicates for the novel axenic assay (with the exception of one compound with two replicates only) and at least two replicates for the intramacrophage assay.

**Fig 6:**
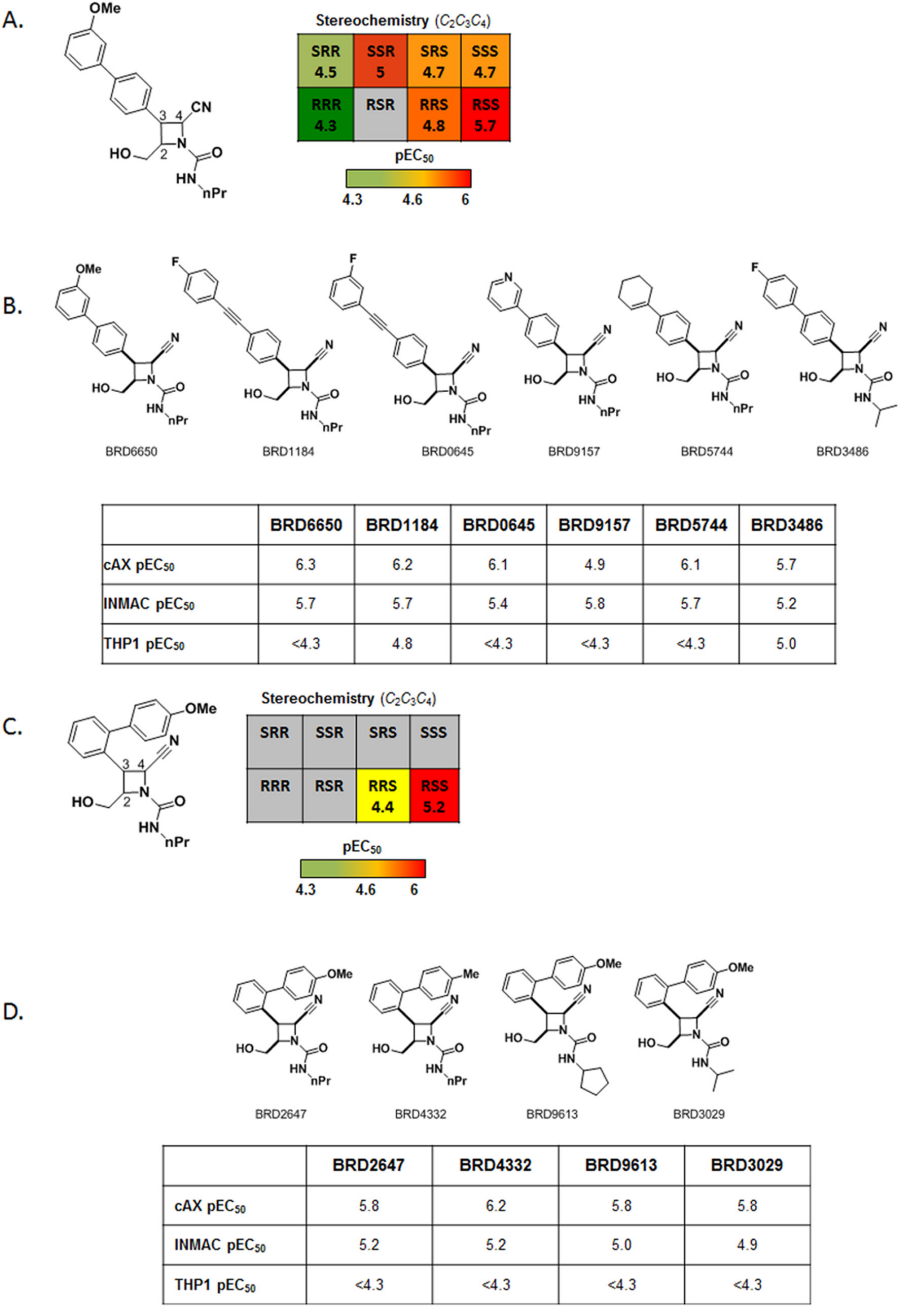
Compound series from the diversity-oriented synthesis library show stereochemistry-dependent selectivity as well as traditional SAR. Compounds from two chemical scaffolds showed reproducible activity in both the novel axenic and intracellular assays. A. Analysis with the Broad's stereochemical structure activity (SSAR) viewer (see Fig E in S1 Text for an example) showed preferential activity with the RSS (C2, C3, C4) stereoisomer of Series 1 compounds. Activity of all available stereoisomers of BRD6650 indicated that the RSS isomer was the most active. B. Analogs of BRD6650 also show SAR around the original hit. Substitution on the phenyl group at the C-3 position of the azetidine ring was tolerated, as was minor variation on the urea functionality (see text for details). C. For compounds from Series 2, preferential activity was observed for the RSS stereoisomer in both thenovel axenic assay screen and the follow-up intracellular assay. D. SAR around both R1 and R2 was also observed for this series; the C-3 phenyl group could be varied with a number of ortho substituents, and variation on the urea was also tolerated.

In order to assess the utility of the screening cascade, a diversity-oriented synthesis library of 9,907 compounds was screened at 25 μM using the novel axenic assay. Compounds that showed a cytocidal effect (i.e. less cells at assay end point compared to assay start point) were retained as primary hits (72 compounds, hit-rate: 0.7%). The primary hits were next profiled using ten-point dose response curves in the novel axenic assay, in a HepG2 toxicity counterscreen and in the intracellular *Leishmania* assay (screening cascade shown in Fig 5A). The confirmation rate in the axenic potency assay was good (62 compounds, or 87% of hits show cidality in retest) and of the confirmed cidal compounds, 57 (92%) showed at least some level of activity in the intracellular assay (percent inhibition at top concentration >50). The potencies obtained in the intracellular assay correlated reasonably well with the axenic data as shown in Fig 5B. Two chemical series in particular showed promising activity in the dose-response retests (Fig 6). An additional 56 related analogues from the DOS library were cherry-picked and tested in dose in the novel axenic, intracellular, and cytotoxicity assays. Stereochemical SAR (SSAR) analysis of all 8 possible isomers of BRD6650 (Series 1, Fig 6 Panels A and B) showed that the RSS stereoisomer was the most potent (intracellular pEC_50_ = 5.7), followed by the SSR stereoisomer (intracellular pEC_50_ = 5). While the SAR around BRD6650 was limited to the compounds tested in the original screening collection, substitution on the phenyl group at the C-3 position of the azetidine ring was tolerated. Both electron-donating and electron-withdrawing groups were active, and diverse functionality was tolerated such as the aryl-alkyne (BRD1184), pyridyl (BRD9157), and cyclohexenyl (BRD5744) groups. Minor variation on the urea functionality was also tolerated. SSAR analysis of Series 2 compounds (e.g. BRD2647, Panels C and D on Fig 6), showed that the RSS stereoisomer was again the most potent stereoisomer (intracellular pEC_50_ = 5.2). The RRS stereoisomer was less potent; the SRR and SSR stereoisomers, which were not tested at dose, were inactive in the initial HTS assay. The other four stereoisomers for this compound were not available. The C-3 phenyl group of BRD2647 could be varied with a number of ortho substituents without loss of potency, and variation on the urea was also tolerated.

## Discussion

The need to identify new candidates entering drug development for visceral leishmaniasis is high considering the low number of compounds currently progressing through the R&D pipeline for this disease [32]. In many disease areas phenotypic screening of large diversity-oriented compound collections has been successful at identifying novel active starting points [8, 9, 33, 34]. For leishmaniasis, finding new start points is hampered by the lack of predictive, high-throughput compatible assays. When compounds are identified, they suffer from the intrinsically high attrition rate associated with early-stage discovery and lead-optimisation programmes [35]. It is therefore critically important to find new approaches to fill the leishmaniasis pipeline. The intramacrophage amastigote *Leishmania* model is currently considered the gold standard for *in vitro* drug susceptibility characterization [17, 18, 22, 36, 37]. Running this assay at a sufficiently high throughput to screen medium- or large-size compound collections is technically as well as financially challenging due to the complexity and limited robustness of this type of assay. While axenic parasites (promastigotes or amastigotes) can be used readily in high-throughput assays, the relatively poor translation into the intracellular assay limits their usefulness [17, 18, 21, 38]. In this work we set out to develop a new *Leishmania in vitro* screening cascade that is suitable for high-throughput screening so that large compound collections can be accessed. As a primary screening platform we developed a new high-throughput axenic amastigote assay with significantly improved predictivity of intramacrophage activity. We have achieved this by altering the assay conditions so that only cytocidal compounds are identified. Our results show that the main reason for the poor correlation between the old axenic assay and our intracellular assay is assay setup related, rather than the result of differences in biology between the different stages (~ 80% of hits identified in our historic axenic assay were not cytocidal). While we cannot be sure this is the case for other published axenic assays as detection limits are rarely reported, we expect this may apply broadly, as a side-effect of using fast growing organisms in combination with relatively low sensitivity read-outs such as resazurin.

In view of the low hit rate for *Leishmania* that we and others have found, there is a concern around potential false negatives. While these clearly exist, many are actually toxic to the host cell in the intracellular assay and can be considered intracellular assay false positives. We have shown that the root cause for approximately half of the non-toxic false-negative compounds that we tested is the difference in concentration that we used to screen in the axenic compared to the intracellular macrophage assay (Fig 2). As most of the remaining false-negatives are weak in the intracellular assay it is possible that they may show activity in the novel axenic assay when tested at higher concentrations.

Interestingly, there are a number of compounds that are active in the novel axenic assay and not active in the intracellular assay. These may represent compounds that hit targets that are not essential intracellularly, perhaps as a result of differences in energy metabolism or supply of essential metabolites. However, our preliminary analysis suggests that these may cluster in a physicochemical space that may preclude their passage through cell membranes under physiological pH. Such compounds may represent novel start points for drug discovery, if their permeability can be improved while maintaining activity.

Testing a panel of previously identified antileishmanials showed good correlation between the novel axenic assay and the intramacrophage assay for most compound classes. The aminoquinolines (mefloquine & amodiaquine) were only active in the intracellular assay, presumably due to their charge at acidic pH, resulting in lack of permeability in the novel axenic assay (pH 5.5) and lysosomotropic accumulation in the intramacrophage assay [39]. Surprisingly, miltefosine was around ten-fold more active in the intracellular assay relative to the novel axenic assay. We and others have previously observed similar activity in axenic and intramacrophage models for miltefosine [17, 18]. Miltefosine is actively taken up by *Leishmania* [40, 41], and therefore it is plausible that the potency difference is due to the much higher cell density in the novel axenic assay compared to our historic assay (80-fold higher), as this could result in lower miltefosine concentrations in the cells and hence lower potency. Disulfiram was only active in the novel axenic assay. Due to technical differences between the two assays there is a higher chance of compounds precipitating in the intracellular assay, and this could have been a problem with disulfiram as its aqueous solubility is poor and in addition this compound is unstable in serum [42]. The activity seen for VL-2098 in the novel axenic assay is in line with recently published data [43], however the activity against intracellular amastigotes is lower than expected from the publication. A second sample of this compound was tested and gave the same results. A potential explanation for this discrepancy is the poor solubility of this compound [44]. Overall the results from this panel of compounds shows that there is a good correlation between our novel axenic assay and our intramacrophage assay, and that almost all compounds with proven antileishmanial activity show activity in the novel axenic assay, supporting its use as a primary screening assay.

We propose that the novel axenic assay is the most suitable primary assay for a screening cascade aimed at accessing large compound collections as it combines the throughput and robustness of axenic assays with good predictivity of intramacrophage activity. Hits from the single-point screen should be tested in potency mode both in the axenic assay and in a human cell counterscreen to rule out any toxicity (ideally targeting a 10-fold or higher selectivity window between the two assays). Finally, the active and selective compounds should be tested in the intracellular model (cascade shown on Fig 4). We validated the use of this cascade by screening a set of ~10,000 DOS compounds and identified two active chemical series of interest. This screen confirmed further that the novel axenic assay is a suitable primary screening platform as 92% of the confirmed actives also showed intracellular activity. The two most interesting hit series incorporate a densely functionalized azetidine ring system each containing three stereogenic elements. While azetidine-related systems such as β-lactams have played an important role in drug discovery, the fully reduced form such as those in our two series of interest have been significantly less studied. These libraries were prepared as part of a collection of skeletally diverse azetidine-based scaffolds. An important feature of this compound collection is that it contains stereochemical diversity and in our experience, Stereochemical Structure Activity (SSAR) analysis of a hit compound can give an indication on how selective and specific its interaction is with its molecular target [45]. While two stereoisomers of BRD6650 were active, the majority of the stereoisomers tested had at least a 9-fold drop in potency indicating a selective and specific interaction with a molecular target. The two series of interest differ in terms of their substituents at R1 and R2 and also the substitution pattern of the phenyl group at C3 which is para in series 1 but ortho in series 2. Synthesis of additional analogues to further investigate SAR and profiling of the current leads in *in vitro* ADME/PK would allow us to assess the potential for these series for therapeutic development.

In summary, we have developed and validated a novel axenic *Leishmania* assay that specifically identifies compounds with a cytocidal mode of action. This new assay has been profiled in terms of its ability to predict activity in the intracellular amastigote *Leishmania* stage which is currently considered the gold standard for *in vitro* drug screening for visceral leishmaniasis. We have demonstrated that this new assay is suitable for primary screening and provides a useful method of triaging compounds to significantly reduce the number of compounds to be profiled and confirmed for activity through the more complex intracellular assays. Additional new active hit series against *Leishmania donovani* are expected to be identified from several ongoing high-throughput screening campaigns incorporating the novel axenic assay as a primary screening tool. Overall, our findings show that assay conditions play a significant role in the nature of the compounds identified (i.e. growth slowing vs cidal) and demonstrate that appropriate axenic assays, particularly in the context of large-scale drug-discovery, can be relevant and of great value.

## Supporting Information

S1 Text Includes the following items: In tabular form-(Table A) Hit-rate and Control definitions in Historic, Novel and Intramacrophage Assay and (Table B) potencies for reference compound panel.In figure form—effect of assay start day (Fig A), different cell stocks (Fig B) and cell passage on assay performance (Fig C); reproducibility of compound potency across assay replicates (Fig D) and a representative plot from the Broad's SSAR viewer tool (Fig E). (DOCX)

## References

[1] WHO. Sustaining the drive to overcome the global impact of neglected tropical diseases: second WHO report on neglected diseases. WHO Library Cataloguing-in-Publication Data. 2013.

[2] Desjeux P. Leishmaniasis. Public health aspects and control. Clinics in dermatology. 1996;14(5):417–23. . 8889319 10.1016/0738-081x(96)00057-0

[3] Desjeux P. Leishmaniasis: current situation and new perspectives. Comparative Immunology, Microbiology and Infectious Diseases. 2004;27(5):305–18. 15225981 10.1016/j.cimid.2004.03.004

[4] Yanik M, Gurel MS, Simsek Z, Kati M. The psychological impact of cutaneous leishmaniasis. Clinical and experimental dermatology. 2004;29(5):464–7. 15347324 10.1111/j.1365-2230.2004.01605.x

[5] McGwire BS, Satoskar AR. Leishmaniasis: clinical syndromes and treatment. QJM: monthly journal of the Association of Physicians. 2014;107(1):7–14. 10.1093/qjmed/hct116 PMC3869292 23744570

[6] Monge-Maillo B, Lopez-Velez R. Therapeutic options for visceral leishmaniasis. Drugs. 2013;73(17):1863–88. 10.1007/s40265-013-0133-0 24170666 PMC3837193

[7] Ready PD. Epidemiology of visceral leishmaniasis. Clinical epidemiology. 2014;6:147–54. 10.2147/CLEP.S44267 24833919 PMC4014360

[8] De Corte BL. From 4,5,6,7-tetrahydro-5-methylimidazo[4,5,1-jk](1,4)benzodiazepin-2(1H)-one (TIBO) to etravirine (TMC125): fifteen years of research on non-nucleoside inhibitors of HIV-1 reverse transcriptase. Journal of medicinal chemistry. 2005;48(6):1689–96. 15771411 10.1021/jm040127p

[9] Keller TH, Shi PY, Wang QY. Anti-infectives: can cellular screening deliver? Current opinion in chemical biology. 2011;15(4):529–33. 10.1016/j.cbpa.2011.06.007 21723774

[10] Callahan HL, Portal AC, Devereaux R, Grogl M. An axenic amastigote system for drug screening. Antimicrob Agents Chemother. 1997;41(4):818–22. 9087496 10.1128/aac.41.4.818 PMC163801

[11] Monte-Alegre A, Ouaissi A, Sereno D. *Leishmania* amastigotes as targets for drug screening. Kinetoplastid Biol Dis. 2006;5:6 17059597 10.1186/1475-9292-5-6 PMC1635419

[12] Shimony O, Jaffe CL. Rapid fluorescent assay for screening drugs on *Leishmania* amastigotes. J Microbiol Methods. 2008;75(2):196–200. 10.1016/j.mimet.2008.05.026 18573286

[13] Debrabant A, Joshi MB, Pimenta PFP, Dwyer DM. Generation of Leishmania donovani axenic amastigotes: their growth and biological characteristics. International Journal for Parasitology. 2004;34(2):205–17. 15037106 10.1016/j.ijpara.2003.10.011

[14] Holzer TR, McMaster WR, Forney JD. Expression profiling by whole-genome interspecies microarray hybridization reveals differential gene expression in procyclic promastigotes, lesion-derived amastigotes, and axenic amastigotes in *Leishmania mexicana* . Mol Biochem Parasitol. 2006;146(2):198–218. Epub 2006/01/25. 10.1016/j.molbiopara.2005.12.009 . 16430978

[15] Li Q, Zhao Y, Ni B, Yao C, Zhou Y, Xu W, et al Comparison of the expression profiles of promastigotes and axenic amastigotes in *Leishmania donovani* using serial analysis of gene expression. Parasitol Res. 2008;103(4):821–8. Epub 2008/06/24. 10.1007/s00436-008-1048-7 18568446

[16] Pescher P, Blisnick T, Bastin P, Spath GF. Quantitative proteome profiling informs on phenotypic traits that adapt *Leishmania donovani* for axenic and intracellular proliferation. Cell Microbiol. 2011. Epub 2011/04/20. 10.1111/j.1462-5822.2011.01593.x 21501362

[17] Vermeersch M, da Luz RI, Tote K, Timmermans JP, Cos P, Maes L. In vitro susceptibilities of *Leishmania donovani* promastigote and amastigote stages to antileishmanial reference drugs: practical relevance of stage-specific differences. Antimicrob Agents Chemother. 2009;53(9):3855–9. Epub 2009/06/24. 10.1128/AAC.00548-09 19546361 PMC2737839

[18] De Rycker M, Hallyburton I, Thomas J, Campbell L, Wyllie S, Joshi D, et al Comparison of a high-throughput high-content intracellular Leishmania donovani assay with an axenic amastigote assay. Antimicrobial agents and chemotherapy. 2013;57(7):2913–22. Epub 2013/04/11. 10.1128/AAC.02398-12 23571538 PMC3697379

[19] Mikus J, Steverding D. A simple colorimetric method to screen drug cytotoxicity against Leishmania using the dye Alamar Blue. Parasitology international. 2000;48(3):265–9. 11227767 10.1016/s1383-5769(99)00020-3

[20] Sereno D, Cordeiro da Silva A, Mathieu-Daude F, Ouaissi A. Advances and perspectives in *Leishmania* cell based drug-screening procedures. Parasitology international. 2007;56(1):3–7. 17079188 10.1016/j.parint.2006.09.001

[21] Siqueira-Neto JL, Song OR, Oh H, Sohn JH, Yang G, Nam J, et al Antileishmanial high-throughput drug screening reveals drug candidates with new scaffolds. PLoS Negl Trop Dis. 2010;4(5):e675 Epub 2010/05/11. 10.1371/journal.pntd.0000675 20454559 PMC2864270

[22] De Muylder G, Ang KK, Chen S, Arkin MR, Engel JC, McKerrow JH. A Screen against *Leishmania* Intracellular Amastigotes: Comparison to a Promastigote Screen and Identification of a Host Cell-Specific Hit. PLoS Negl Trop Dis. 2011;5(7):e1253 Epub 2011/08/04. 10.1371/journal.pntd.0001253 21811648 PMC3139667

[23] Rosenzweig D, Smith D, Opperdoes F, Stern S, Olafson RW, Zilberstein D. Retooling Leishmania metabolism: from sand fly gut to human macrophage. FASEB journal: official publication of the Federation of American Societies for Experimental Biology. 2008;22(2):590–602. 17884972 10.1096/fj.07-9254com

[24] Burchmore RJ, Barrett MP. Life in vacuoles—nutrient acquisition by Leishmania amastigotes. International journal for parasitology. 2001;31(12):1311–20. Epub 2001/09/22. 11566299 10.1016/s0020-7519(01)00259-4

[25] De Rycker M, O'Neill S, Joshi D, Campbell L, Gray DW, Fairlamb AH. A static-cidal assay for Trypanosoma brucei to aid hit prioritisation for progression into drug discovery programmes. PLoS Negl Trop Dis. 2012;6(11):e1932 Epub 2012/12/05. 10.1371/journal.pntd.0001932 23209868 PMC3510075

[26] Dandapani S, Marcaurelle LA. Grand challenge commentary: Accessing new chemical space for 'undruggable' targets. Nature chemical biology. 2010;6(12):861–3. 10.1038/nchembio.479 21079589

[27] Brenk R, Schipani A, James D, Krasowski A, Gilbert IH, Frearson J, et al Lessons learnt from assembling screening libraries for drug discovery for neglected diseases. ChemMedChem. 2008;3(3):435–44. Epub 2007/12/08. 18064617 10.1002/cmdc.200700139 PMC2628535

[28] Lowe JT, Lee MDt, Akella LB, Davoine E, Donckele EJ, Durak L, et al Synthesis and profiling of a diverse collection of azetidine-based scaffolds for the development of CNS-focused lead-like libraries. The Journal of organic chemistry. 2012;77(17):7187–211. 10.1021/jo300974j 22853001 PMC3454511

[29] Marcaurelle LA, Comer E, Dandapani S, Duvall JR, Gerard B, Kesavan S, et al An aldol-based build/couple/pair strategy for the synthesis of medium- and large-sized rings: discovery of macrocyclic histone deacetylase inhibitors. Journal of the American Chemical Society. 2010;132(47):16962–76. 10.1021/ja105119r 21067169 PMC3004530

[30] Lovering F, Bikker J, Humblet C. Escape from flatland: increasing saturation as an approach to improving clinical success. Journal of medicinal chemistry. 2009;52(21):6752–6. 10.1021/jm901241e 19827778

[31] Patterson S, Wyllie S, Stojanovski L, Perry MR, Simeons FR, Norval S, et al The R enantiomer of the antitubercular drug PA-824 as a potential oral treatment for visceral Leishmaniasis. Antimicrob Agents Chemother. 2013;57(10):4699–706. 10.1128/AAC.00722-13 23856774 PMC3811480

[32] DNDi. DNDi R&D Projects. http://wwwdndiorg/diseases-projects/portfoliohtml.

[33] Duffy KJ, Darcy MG, Delorme E, Dillon SB, Eppley DF, Erickson-Miller C, et al Hydrazinonaphthalene and azonaphthalene thrombopoietin mimics are nonpeptidyl promoters of megakaryocytopoiesis. Journal of medicinal chemistry. 2001;44(22):3730–45. 11606138 10.1021/jm010283l

[34] Yamamura Y, Ogawa H, Chihara T, Kondo K, Onogawa T, Nakamura S, et al OPC-21268, an orally effective, nonpeptide vasopressin V1 receptor antagonist. Science. 1991;252(5005):572–4. 1850553 10.1126/science.1850553

[35] Don R, Ioset JR. Screening strategies to identify new chemical diversity for drug development to treat kinetoplastid infections. Parasitology. 2014;141(1):140–6. Epub 2013/08/30. 10.1017/S003118201300142X 23985066

[36] Siqueira-Neto JL, Moon S, Jang J, Yang G, Lee C, Moon HK, et al An Image-Based High-Content Screening Assay for Compounds Targeting Intracellular *Leishmania donovani* Amastigotes in Human Macrophages. Plos Neglect Trop D. 2012;6(6):e1671. Epub 2012/06/22. 10.1371/journal.pntd.0001671 PMC3373640 22720099

[37] Aulner N, Danckaert A, Rouault-Hardoin E, Desrivot J, Helynck O, Commere PH, et al High Content Analysis of Primary Macrophages Hosting Proliferating Leishmania Amastigotes: Application to Anti-leishmanial Drug Discovery. Plos Neglect Trop D. 2013;7(4). 10.1371/journal.pntd.0002154 PMC3617141 23593521

[38] Ephros M, Bitnun A, Shaked P, Waldman E, Zilberstein D. Stage-specific activity of pentavalent antimony against Leishmania donovani axenic amastigotes. Antimicrob Agents Chemother. 1999;43(2):278–82. 9925518 10.1128/aac.43.2.278 PMC89063

[39] Naisbitt DJ, Ruscoe JE, Williams D, O'Neill PM, Pirmohamed M, Park BK. Disposition of amodiaquine and related antimalarial agents in human neutrophils: implications for drug design. The Journal of pharmacology and experimental therapeutics. 1997;280(2):884–93. 9023303

[40] Perez-Victoria FJ, Sanchez-Canete MP, Castanys S, Gamarro F. Phospholipid translocation and miltefosine potency require both L. donovani miltefosine transporter and the new protein LdRos3 in Leishmania parasites. The Journal of biological chemistry. 2006;281(33):23766–75. 16785229 10.1074/jbc.M605214200

[41] Perez-Victoria FJ, Gamarro F, Ouellette M, Castanys S. Functional cloning of the miltefosine transporter. A novel P-type phospholipid translocase from Leishmania involved in drug resistance. The Journal of biological chemistry. 2003;278(50):49965–71. 14514670 10.1074/jbc.M308352200

[42] Cobby J, Mayersohn M, Selliah S. The rapid reduction of disulfiram in blood and plasma. The Journal of pharmacology and experimental therapeutics. 1977;202(3):724–31. 197231

[43] Gupta S, Yardley V, Vishwakarma P, Shivahare R, Sharma B, Launay D, et al Nitroimidazo-oxazole compound DNDI-VL-2098: an orally effective preclinical drug candidate for the treatment of visceral leishmaniasis. The Journal of antimicrobial chemotherapy. 2015;70(2):518–27. 10.1093/jac/dku422 25389223

[44] Mukkavilli R, Pinjari J, Patel B, Sengottuvelan S, Mondal S, Gadekar A, et al In vitro metabolism, disposition, preclinical pharmacokinetics and prediction of human pharmacokinetics of DNDI-VL-2098, a potential oral treatment for Visceral Leishmaniasis. Eur J Pharm Sci. 2014;65:147–55. Epub 2014/09/28. 10.1016/j.ejps.2014.09.006 25261338

[45] Mulrooney CA, Lahr DL, Quintin MJ, Youngsaye W, Moccia D, Asiedu JK, et al An informatic pipeline for managing high-throughput screening experiments and analyzing data from stereochemically diverse libraries. Journal of computer-aided molecular design. 2013;27(5):455–68. 10.1007/s10822-013-9641-y 23585218 PMC5554947

