# Supplementary material for: Development and Validation of a Novel Leishmania donovani Screening Cascade for High-Throughput Screening Using a Novel Axenic Assay with High Predictivity of Leishmanicidal Intracellular Activity
Source: PLoS Negl Trop Dis. 2015 Sep 25;9(9):e0004094. doi: 10.1371/journal.pntd.0004094 (PMC4583543; doi:10.1371/journal.pntd.0004094)
Supplement: S1 Text — (DOCX) [file pntd.0004094.s001.docx]

**Supplementary Tables**

**Table A. Hit-rate and Control definitions in Historic, Novel and Intramacrophage Assay.**

|  | **0** **% Inhibition control** | **100** **% Inhibition control** | **0** **% THP-1 inhibition** | **Hit-rate definition** |
| --- | --- | --- | --- | --- |
| **Historic axenic** | DMSO | media only | N/A | > 70 % inhibition |
| **Novel axenic** | axenic amastigotes at starting density | 2 µM Amphotericin B | N/A | > 0 % inhibition |
| **Intramacrophage** | DMSO | 2 µM Amphotericin B | Infected THP-1 cells / DMSO | > 70 % inhibition of intracellular amastigotes |

**Table B. Potencies for reference compound panel.**

| **Compound Name** | **Class** | **Novel** | **Intramacrophage** | **THP-1** | **HepG2** |
| --- | --- | --- | --- | --- | --- |
| Imiquimod | imidazoquinolinamine | < 4.3 | < 4.46 | < 4.46 | < 4.3 |
| VL-0614 | aminothiazoles | 5.87 (0.25) | 5.2 (0.25) | 4.46 (0.01) | N/A |
| VL-0672 | aminothiazoles | 5.96 (0.16) | 5.42 (0.22) | 4.67 (0.47) | N/A |
| VL-2098 | nitroimidazoles | 7.75 (0.5) | 5.78 (0.07) | < 4.46 | N/A |
| VL-0505 | aminothiazoles | 6.15 (0.12) | 5.86 (0.05) | 4.52 (0.1) | N/A |
| 35501 | nitroimidazoles | 6.6 (0.11) | 5.75 (0.06) | 4.5 (0.1) | N/A |
| 35711 | nitroimidazoles | 6.24 (0.19) | 5.68 (0.14) | < 4.46 | N/A |
| 35781 | nitroimidazoles | 6.24 (0.24) | 5.65 (0.16) | < 4.46 | N/A |
| AN10398 | oxaboroles | 6.02 (0.16) | 5.45 (0.33) | < 4.46 | N/A |
| AN9958 | oxaboroles | 5.8 (0.05) | 5.63 (0.19) | 4.98 (0.72) | N/A |
| AN10199 | oxaboroles | 5.92 (0.15) | 5.38 (0.13) | 4.47 (0.04) | N/A |
| Docetaxel | taxols | 5.05 (0.35) | 4.84 (0.43) | < 4.46 | N/A |
| Miltefosine | alkylphospholipids | 4.5 (0.36) | 6.1 (0.24) | < 4.46 | < 4.3 |
| Fexinidazole sulfone | nitroimidazoles | 5.11 (0.18) | 4.49 (0.08) | < 4.46 | < 4.3 |
| Amphotericin B | polyenes | 6.52 (0.19) | 6.62 (0.2) | 4.57 (0.28) | < 4.3 |
| Pyrazinamide | carboxamides | < 4.3 | < 4.46 | < 4.46 | < 4.3 |
| Nystatin | polyenes | 5.19 (0.21) | 4.68 (0.5) | < 4.46 | < 4.3 |
| Posaconazole | azoles | 4.44 (0.25) | 4.73 (0.42) | 4.75 (0.46) | 5.07 (0.17) |
| Paromomycin | aminoglycosides | < 4.3 | < 4.46 | < 4.46 | < 4.3 |
| Clemastine | ethanolamines | < 4.3 | 4.8 (0.2) | 4.75 (0.12) | 4.66 (0.01) |
| Disulfiram | disulfurs | 6.44 (0.11) | < 4.46 | < 4.46 | 4.34 (0.03) |
| Nifurtimox | nitrofurans | 4.95 (0.19) | < 4.46 | < 4.46 | < 4.3 |
| Cantharidin | cantharidins | 5.36 (0.17) | 4.88 (0.47) | 5.56 (0.08) | 5.87 (0.07) |
| Suramin | suramins | < 4.3 | < 4.46 | < 4.46 | < 4.3 |
| Fenarimol | fenarimols | < 4.3 | < 4.46 | < 4.46 | < 4.3 |
| Pentamidine | diamidines | 5.06 (0.23) | 4.81 (0.22) | < 4.46 | 5.55 (0.23) |
| Menadione | naphthoquinones | 4.99 (0.25) | < 4.46 | 4.48 (0.05) | 5.46 (0) |
| Luteolin | flavonoids | 4.33 (0.05) | 4.55 (0.09) | 4.84 (0.12) | 4.61 (0.24) |
| Berberine | alkaloids | 5.03 (0.17) | 4.79 (0.17) | < 4.46 | 4.47 (0.12) |
| Indinavir | piperazines | < 4.3 | < 4.46 | < 4.46 | < 4.3 |
| Amodiaquine | 4-aminoquinolines | < 4.3 | 5.39 (0.09) | 4.53 (0.05) | 4.59 (0.12) |
| Mefloquine | 4-aminoquinolines | < 4.3 | 5.07 (0.43) | 4.92 (0.44) | 5.01 (0.12) |
| Alendronic acid | biphosphonates | < 4.3 | < 4.46 | < 4.46 | < 4.3 |
| Licochalcone A | chalcones | 4.67 (0.15) | 4.65 (0.17) | 4.46 (0.01) | 4.44 (0.09) |
| Diminazene | diamidines | 4.93 (0.24) | < 4.46 | < 4.46 | < 4.3 |
| Fexinidazole | nitroimidazoles | 4.94 (0.2) | < 4.46 | < 4.46 | < 4.3 |

Numbers in brackets indicate the standard deviation.

**Supplementary Figures**

Fig A.


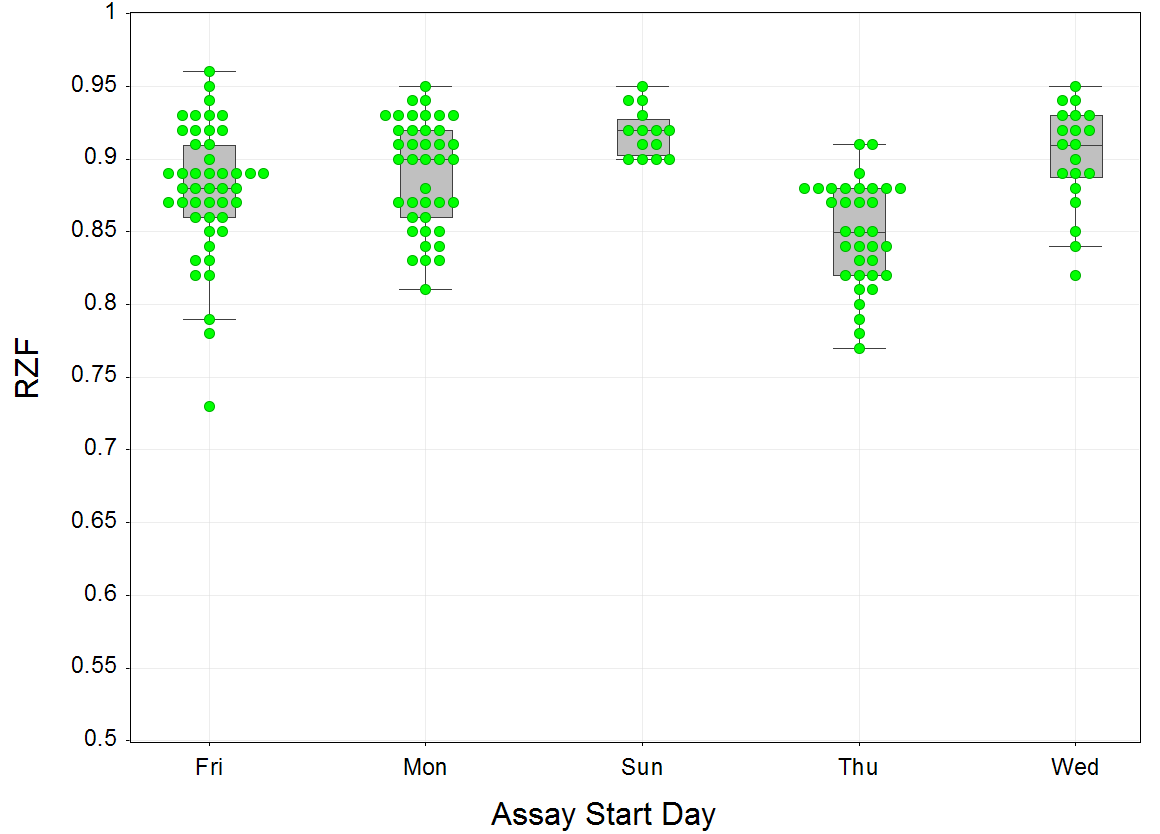


Fig A. Effect of assay start day on assay performance. Boxplot showing plate robust Z-factor (RZF) by assay start day. Data set represents 154 individual data-points in total (shown in green; number of plates for each day: Monday: 40, Wednesday: 20, Thursday 34, Friday: 46 and Sunday 14).

Fig B.


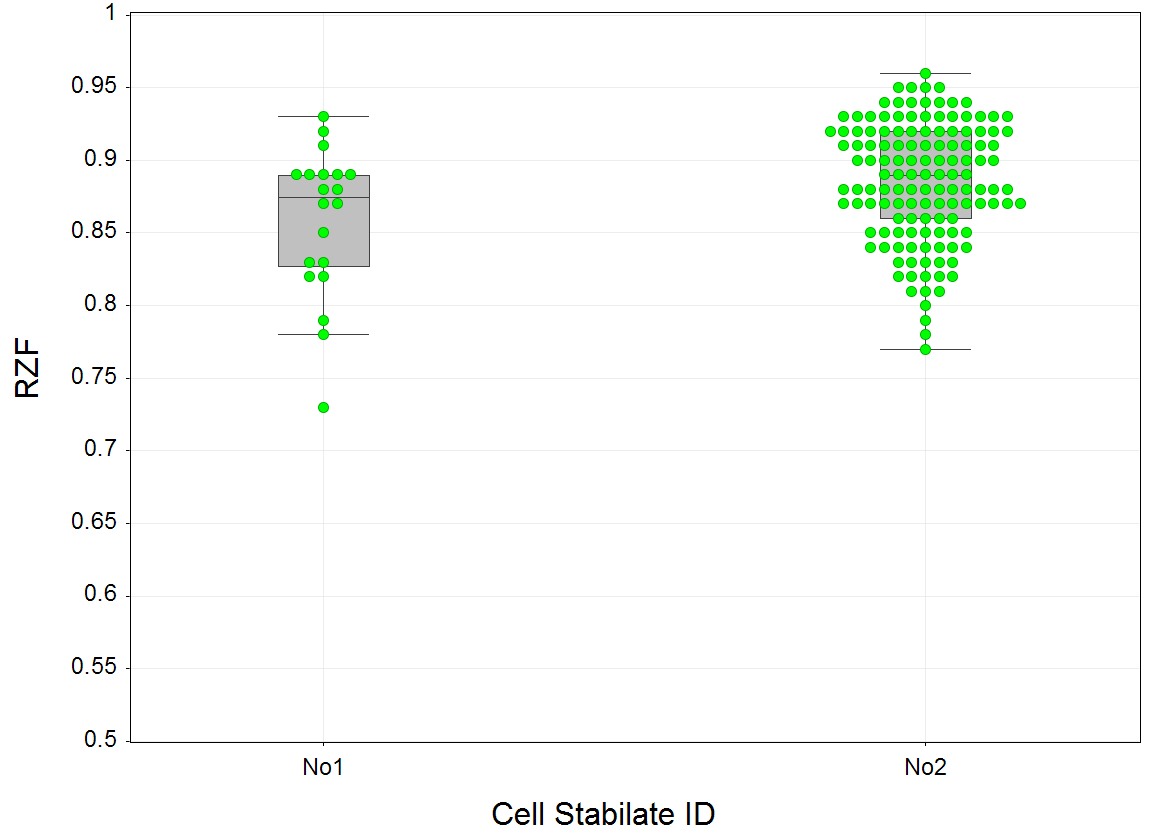
Fig B. Effect of cell stock aliquot on assay performance. This boxplot shows two different cell stocks (derived from different frozen aliquots of the same strain) against the RZF. Data set represents 154 individual data points (shown in green) with 20 assay plates started with cell stock no 1 and 134 assay plates started with cell stock no 2.

Fig C.


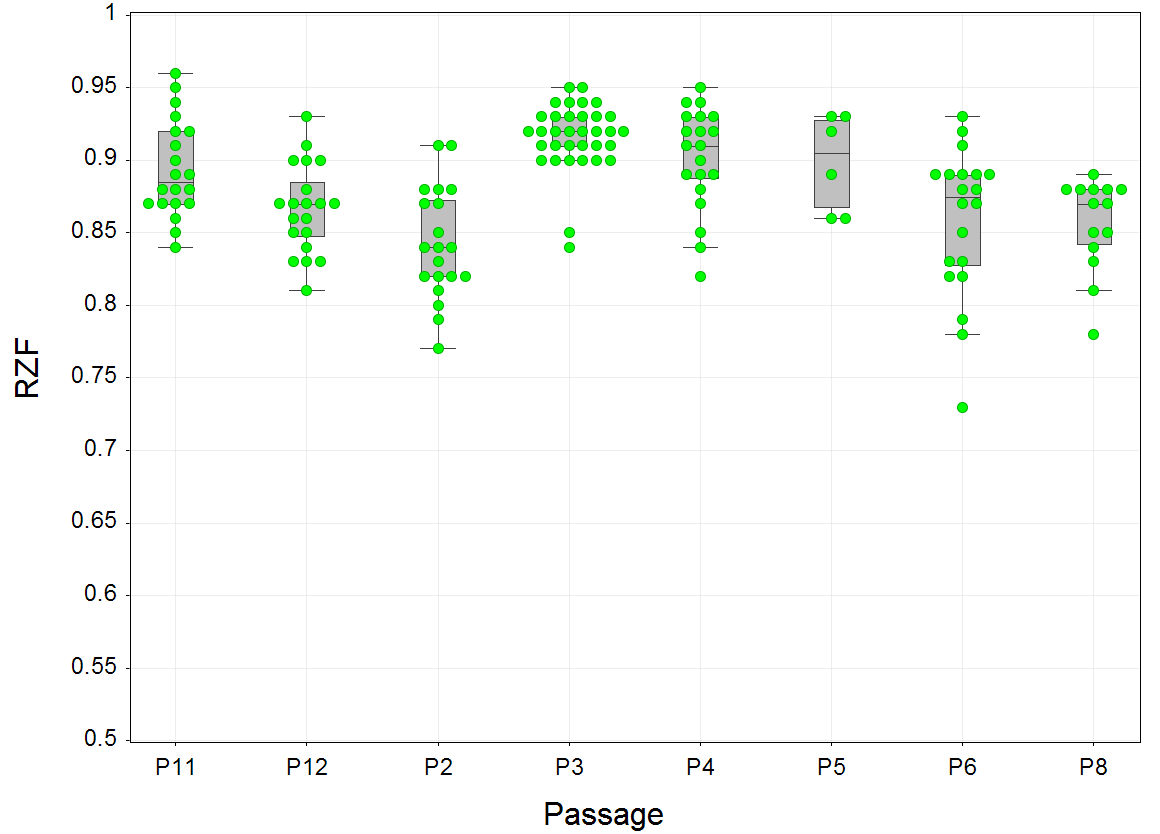


Fig C. Effect of cell passage number (P) on assay performance. This boxplot shows different cell passage numbers against the RZF. Data set represents 154 individual data points in total. Cell passage is counted after conversion from promastigote form to amastigote form. Number of plates for each passage is as follows: 20 for P2, P6, P4, P11 and P12, 34 for P3, 6 for P5 and 14 for P8.

Fig D.


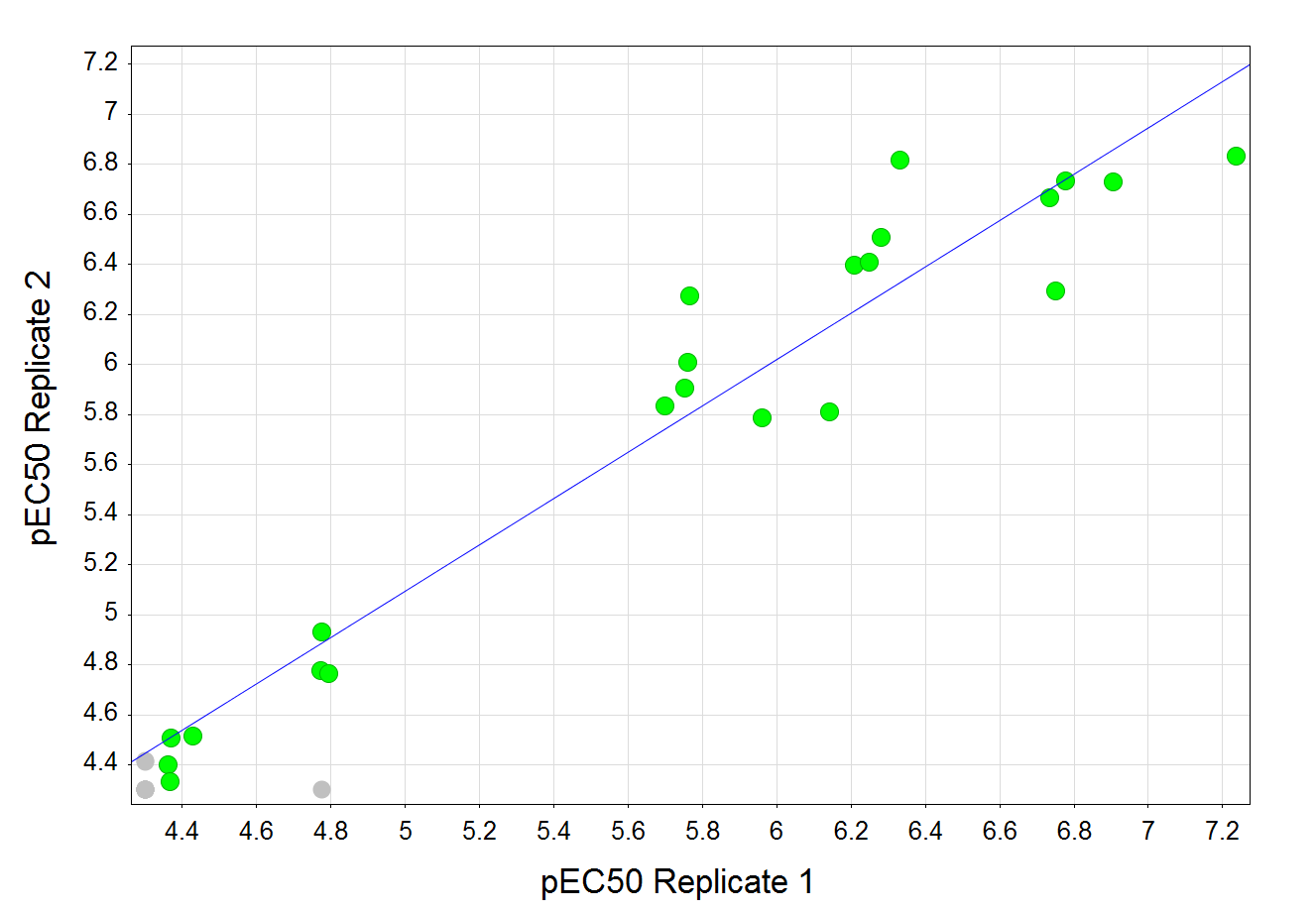
Fig D. Novel axenic assay potency replicates. The potencies of two biological replicates for 35 compounds are plotted. Blue line shows linear regression (R^2^ = 0.95).

Fig E


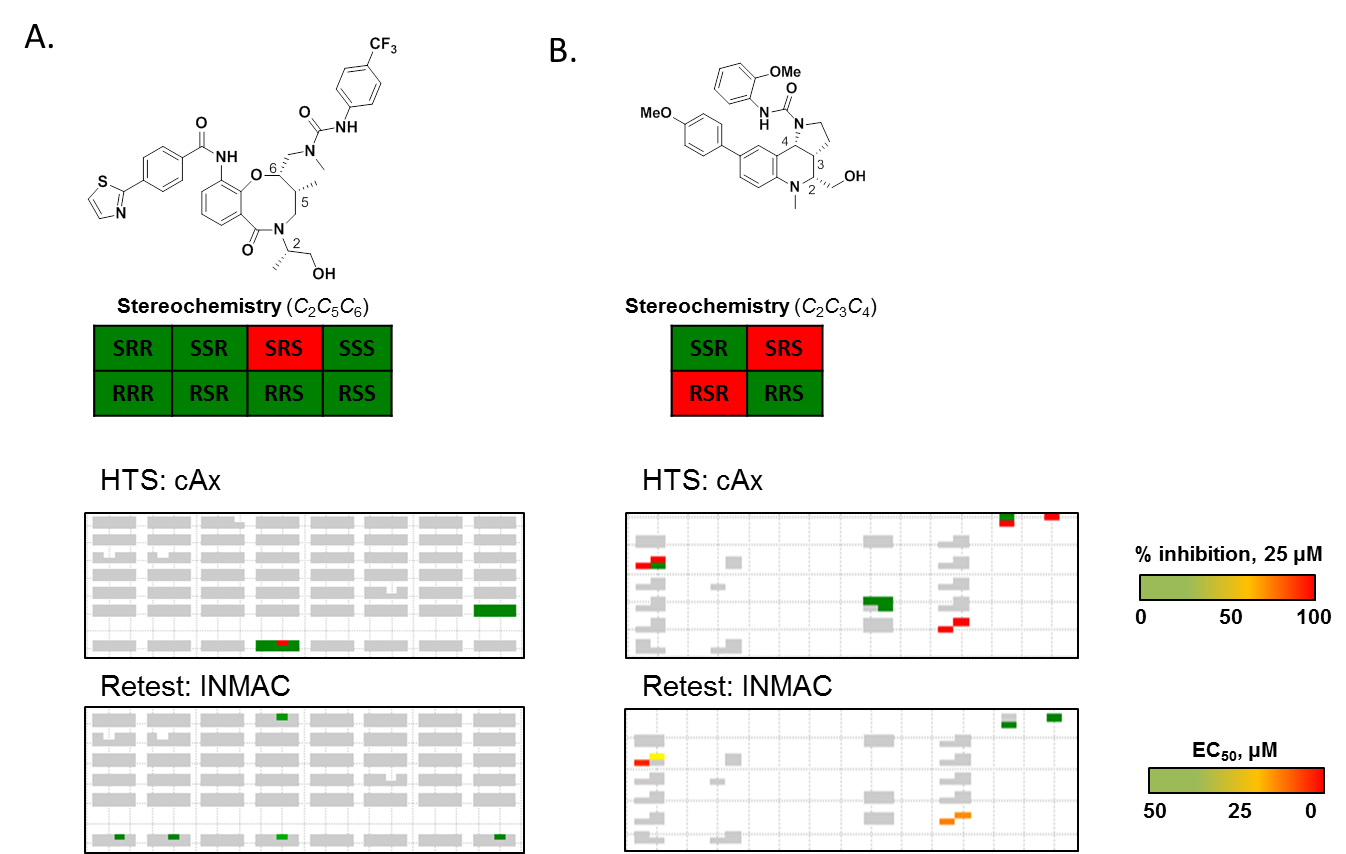


Fig E. cAx high throughput and intracellular dose response data representations in the Broad's SSAR viewer tool**.** Representative hit compounds and active stereoisomers are shown at the top of each panel. The boxes below show snapshots of the library data for the indicated assay. Each box represents a set of stereoisomers for the given scaffold, e.g. 8 possible stereoisomers in Panel A and 4 possible stereoisomers for the compound shown in Panel B. Grey boxes indicate compounds that were not screened, and white areas indicate compounds that were not synthesized. The activity in each assay is color coded as shown in the key on the right, with more active compounds shown in red. **A.** Compounds from the SnAr8-ortho library showed stereoselectivity in the cAx HTS assay, with only the SRS compound showing activity. Follow-up intracellular assays showed weak activity of the original hit in the intracellular assay (pEC_50_ = 4.4) and better activity in the cAx assay (pEC_50_ = 4.6, data not shown). Additional analogues tested at dose showed minimal activity in the intracellular assay. **B.** Compounds from the Povarov library were more potent in both the cAx and intracellular dose assays. However, as shown in the SSAR view, there were several instances of enantiomers with very similar activity, suggesting that stereochemistry is not a component of the activity for this scaffold.
